# West Nile Virus and Usutu Virus Monitoring of Wild Birds in Germany

**DOI:** 10.3390/ijerph15010171

**Published:** 2018-01-22

**Authors:** Friederike Michel, Dominik Fischer, Martin Eiden, Christine Fast, Maximilian Reuschel, Kerstin Müller, Monika Rinder, Sylvia Urbaniak, Florian Brandes, Rebekka Schwehn, Renke Lühken, Martin H. Groschup, Ute Ziegler

**Affiliations:** 1 Friedrich-Loeffler Insitut (FLI), Federal Research Institute for Animal Health, Institute of Novel and Emerging Infectious Diseases, Südufer 10, D-17493 Greifswald-Insel Riems, Germany; friederike.michel@fli.de (F.M.); martin.eiden@fli.de (M.E.); christine.fast@fli.de (C.F.); martin.groschup@fli.de (M.H.G.); 2 Clinic for Birds, Reptiles, Amphibians and Fish, Justus Liebig University Giessen, Frankfurter Straße 91, D-35392 Giessen, Germany; dominik.fischer@vetmed.uni-giessen.de; 3 Clinic for Small Mammals, Reptiles and Birds, University of Veterinary Medicine Hannover, Foundation, Bünteweg 9, D-30559 Hannover, Germany; maximilian.reuschel@tiho-hannover.de; 4 Department of Veterinary Medicine, Small Animal Clinic, Freie Universität Berlin, Oertzenweg 19 b, D-14163 Berlin, Germany; Kerstin.Mueller@fu-berlin.de; 5 Clinic for Birds, Small Mammals, Reptiles and Ornamental Fish, Centre for Clinical Veterinary Medicine, Ludwig Maximilians University Munich, Sonnenstraße 18, D-85764 Oberschleißheim, Germany; monika.rinder@vogelklinik.vetmed.uni-muenchen.de; 6 Birds of Prey Rehab Center Rhineland (Greifvogelhilfe Rheinland), Roermonder Straße 34, D-41379 Brüggen, Germany; info@greifvogelhilfe.de; 7 Wildtier-und Artenschutzstation, Hohe Warte 1, D-31553 Sachsenhagen, Germany; florian.brandes@wildtierstation.de; 8 Seehundstation Nationalpark-Haus Norden-Norddeich, Dörper Weg 24, D-26506 Norden, Germany; rebekka.schwehn@gmail.com; 9 Bernhard-Nocht-Institute for Tropical Medicine, WHO Collaborating Centre for Arbovirus and Hemorrhagic Fever Reference and Research, Bernhardt-Nocht Straße 74, D-20359 Hamburg, Germany; luehken@bnitm.de

**Keywords:** West Nile virus, Usutu virus, wild bird, monitoring, network, Germany

## Abstract

By systematically setting up a unique nation-wide wild bird surveillance network, we monitored migratory and resident birds for zoonotic arthropod-borne virus infections, such as the flaviviruses West Nile virus (WNV) and Usutu virus (USUV). More than 1900 wild bird blood samples, from 20 orders and 136 different bird species, were collected between 2014 and 2016. Samples were investigated by WNV and USUV-specific real-time polymerase chain reactions as well as by differentiating virus neutralization tests. Dead bird surveillance data, obtained from organ investigations in 2016, were also included. WNV-specific RNA was not detected, whereas four wild bird blood samples tested positive for USUV-specific RNA. Additionally, 73 USUV-positive birds were detected in the 2016 dead bird surveillance. WNV neutralizing antibodies were predominantly found in long-distance, partial and short-distance migrants, while USUV neutralizing antibodies were mainly detected in resident wild bird species, preferentially with low seroprevalences. To date, WNV-specific RNA has neither been detected in wild birds, nor in mosquitoes, thus, we conclude that WNV is not yet present in Germany. Continued wild bird and mosquito monitoring studies are essential to detect the incursion of zoonotic viruses and to allow risk assessments for zoonotic pathogens.

## 1. Introduction

Wild birds play an important role as reservoir hosts and as a transport shuttle for zoonotic arboviruses and their arthropod hosts to Central Europe. In particular, migratory birds play an important role in the spread of novel viruses to new areas along the major wild bird flyways across Asia, Africa, and Europe. Therefore, monitoring studies in wild birds can be used as an early warning system for the incursion of a number of zoonotic pathogens. We have monitored the infection and seroprevalence status of migratory and resident birds for many years, and have set up a German nationwide wild bird surveillance network for zoonotic arthropod-borne virus infections, with special emphasis on zoonotic flaviviruses, such as West Nile virus (WNV) and Usutu virus (USUV).

WNV is an arthropod-borne, single-stranded RNA virus, belonging to the family *Flaviviridae* [1], and is considered to be the most widespread flavivirus in the world [2,3]. The virus circulates between mosquitoes, which act as vectors, and wild birds, which act as reservoir hosts [4]. The composition of bird and mosquito species differs between the geographical regions with WNV circulation [5]. Wild birds serve as amplifying hosts; they develop a strong and long-term viremia, and are capable of infecting bird-biting mosquitoes [6]. As a rule, most birds undergo subclinical infection and do not develop clinical symptoms [7]. However, there are some highly-susceptible bird species, such as birds of prey, jays, and crows, which have been shown to develop severe and usually fatal encephalitis [6,7,8]. As shown by Nehmeth et al. [9], persistently WNV-infected birds can act as carriers between WNV endemic areas, and distribute WNV to new, previously disease-free areas. As every year millions of wild birds migrate between Europe and Africa, they can act as an entry portal, by overwintering in, or passing through, WNV-endemic areas [10,11].

Infection of susceptible non-avian vertebrates is usually asymptomatic, but, humans and horses in particular, can develop disease as a consequence of WNV infections, which may range from mild febrile illness (West Nile fever) to encephalitis with fatal outcome [12,13,14]. In Europe, several countries, such as Ukraine, Romania, Russia, France, Italy, and Hungary, have reported WNV infections in humans and horses during the last decades [2,15,16,17]. In the last few years, WNV cases have been observed, particularly in Southern Europe, often associated with the major flyways of migratory birds [18]. In previous wild bird studies in Germany, from 2007 to 2013, neutralizing antibodies against WNV could be detected, primarily in migratory birds, but WNV-specific RNA has not been found yet [19,20,21,22].

USUV is a close relative of WNV, which was probably introduced to Europe (Italy) in 1996 [23,24]. The first large outbreak of USUV occurred in 2001, in Austria, with a significant die-off of Eurasian Blackbirds (*Turdus merula*) and Great Grey Owls (*Strix nebulosa*) [25]. Since then, the virus has spread to different European countries, such as Hungary, Switzerland, Spain, Belgium, Czech Republic, France, and Croatia [26,27,28]. In 2010, USUV was isolated from a pool of *Culex pipiens pipiens* mosquitoes in Southwestern Germany [29]. The following two years (2011/12), USUV caused a massive die-off in Eurasian Blackbirds in the Upper Rhine valley [20,30], resulting in a continuous decline of the species population in USUV-suitable areas [31]. The German Federal States Rhineland-Palatinate, Baden-Wuerttemberg, and Hesse were the major distribution areas for USUV, and sporadic cases were also observed in Cologne and Bonn. Between 2013 and 2015, the number of USUV positive birds decreased. In 2016, a dramatic increase in the number of USUV positive birds was detected. Besides the known USUV epidemic areas spanning the Upper Rhine valley, a cumulative emergence of USUV in the Northern parts of North-Rhine Westphalia up to the border to The Netherlands has been found as well as a higher occurrence of USUV positive birds in the region of Leipzig. Surprisingly, the causative USUV strains found in 2016 represented four lineages, of which two putative novel lineages most likely have been introduced into Germany just recently [32,33], from where they probably have spread to other western European countries causing mass-die-offs, preferentially in Eurasian Blackbirds in The Netherlands and Belgium [33,34,35,36]. While USUV originally was considered as an arbovirus with low zoonotic potential, recent data from various European countries indicate that there also might be a much higher number of clinical neuroinvasive USUV infections in humans than assumed to date [37,38].

Whereas USUV has been endemic in Germany since 2010, WNV has not been detected so far, but an incursion is possible. A spreading tendency of the virus in the Northern direction is already apparent, and vectors and hosts are already present. This study continued the molecular and serological surveillance for WNV and USUV in wild birds in Germany, which has been going since 2014. This is the first time that such an extensive monitoring study for WNV and USUV in wild birds with different collection sites distributed all over Germany has been carried out. As WNV and USUV have similar transmission cycles between birds as main amplifying hosts and mosquitoes as vectors, co-circulation as well as overlapping transmission cycles cannot be excluded.

## 2. Materials and Methods 

### 2.1. Sample Collection

**Figure 1:**
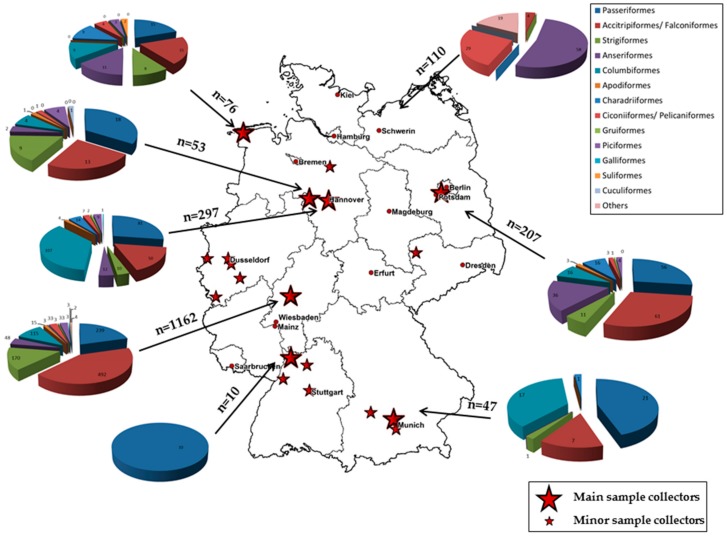
Location of sampling sites with zoological orders of wild bird blood samples from 2014–2016 (big red stars = main collectors, small red stars = minor collectors). The samples highlighted in the pie chart represent the total of all samples collected by all minor and major collectors in each region of Germany.

We monitored migratory and resident birds by systematically setting up a nation-wide wild bird surveillance network for zoonotic arthropod-borne virus infections. This unique German monitoring network, within the frame of the German Centre for Infection Research (DZIF), includes 22 different collection sites, distributed all over Germany, and brings together veterinary universities or institutes, bird clinics, wild bird rescue stations, zoological gardens, as well as ornithologists. Between 2014 and 2016, 1962 blood samples from resident and migratory birds belonging to 136 different bird species, in 20 bird orders, were collected (see Table 1). Birds were categorized as resident birds (stay all year round in their German habitat), partial migratory birds (parts of the population stay in the German habitat and parts migrate), short-distance migratory birds (usually migrating 1000–2000 km and not passing the Sahara desert), and long-distance migratory birds (usually migrating ≥3000–4000 km and/or passing the Sahara desert). The geographic distribution of the sampling sites and the zoological orders of examined wild birds are depicted in Figure 1. During the study, extra marking of sampled birds was not allowed by the competent authority. However, if birds had been banded or marked in another way previously, this was documented, in order to detect recaptures of individuals. Moreover, radiographs, weight and pictures of non-marked birds of the same species were compared, in order to differentiate individuals. Therefore, double recording of data from the same bird is unlikely, but cannot be excluded completely. Birds were bled by puncturing their wing veins or jugular veins, and, after blood separation, cruor was stored at −70 °C and sera at −20 °C and were processed individually. These samples were analyzed with WNV- and USUV-specific quantitative real-time polymerase chain reactions (qRT-PCR) and virus-specific neutralization tests (VNT). In rare cases, where the collection of both cruor and serum was not possible, the analysis was restricted to either qRT-PCR or VNT.

In addition, in 2016, we obtained organ samples from over 100 diseased or dead wild birds which were submitted by bird clinics, local state veterinary laboratories, zoological gardens, or bird rehabilitation centers, or which were collected by assistants of the German Mosquito Control Association or Nature and Biodiversity Conservation Union and screened in cooperation with the Bernhard Nocht Institute for Tropical Medicine and the Institute of Virology Leipzig (Faculty of Veterinary Medicine).

### 2.2. Ethics Statement

Blood samples were taken during routine clinical examination of injured, diseased or orphaned wild birds which had been admitted to different bird clinics, bird veterinarians, or wild bird rescue centers. Leftover blood material from the birds was used for this project.

### 2.3. Real-Time RT-PCR

Viral RNA from the avian blood samples was isolated from cruor using the RNeasy Mini Kit (Qiagen, Hilden, Germany), according to the manufacturer’s instructions. Extracted RNA was detected in a WNV-specific qRT-PCR [29]. Primer and probes were targeted to the 5′untranslated region (UTR), which enables simultaneous detection of WNV lineages 1 and 2 [39]. Furthermore, the USUV-specific qRT-PCR was performed using the protocol described by Jöst et al. [29]. Cycle threshold (Ct) values below 37 were regarded as positive, from 37 to 40 as suspicious, and above 40 as negative. The same procedure was applied to the tissues (brain, liver, spleen or heart; depending on availability) from the dead birds after dissection.

### 2.4. Serological Investigations

In the absence of a suitable ELISA for detection of WNV and USUV antibodies in birds requiring only small sample volumes (<50 μL), and an ELISA with a high sensitivity and specificity against the serogroup cross-reactivity with other flaviviruses, we decided to investigate all 1825 serum samples from 136 different species of resident and wild birds by using differentiating virus neutralization tests. At first, all serum samples were screened for WNV- and USUV-specific antibodies in two dilution steps (1:10 and 1:20), and only positive samples were diluted further to determine endpoint titers.

We analyzed all wild bird serum samples against WNV strain Austria (acc. no. HM015884, kindly provided by S. Revilla-Fernandez, AGES Mödlingen, Austria) and USUV strain Germany (acc. no. HE599647) in specific VNTs to identify the cross-reacting antibody reactions among the Japanese encephalitis serogroup. A small number of samples could not be evaluated, due to hemolysis and cytotoxic effects on the cells or due to low sample volumes. VNT was performed, as described by Seidowski et al. (2010) and Ziegler et al. (2015) [19,20]. All samples were run in duplicate and in a final serum dilution of 1:10 and a virus concentration of 100 TCID_50_/well. Cytopathic effects were seen 6–7 days post infection, and the neutralizing antibody titers (ND_50_) were calculated according to the Behrens–Kaerber method. Serum samples with ND_50_ values above 10 were determined to be positive; samples with a lower titer than 10 were determined to be negative. The heat-inactivated serum samples were tested individually, at two independent times, in both WNV- and USUV-specific VNTs, given that enough serum was available.

The birds that tested positive for WNV antibodies had negative (ND_50_ < 10) or significantly lower (on average two-fold lower) USUV titers. The same applied to birds that tested positive for USUV antibodies which showed negative or significantly lower WNV titers.

## 3. Results

We investigated 1902 blood samples for WNV- and USUV-specific RNA. WNV-specific RNA was not detected in any of the samples, whereas four live wild birds were positive for USUV-specific genome sequences by qRT-PCR. In detail, in 2014, one Eurasian Blackbird from Giessen, in the federal state Hesse, in 2015, one Eurasian Blackbird and one City Pigeon (*Columba livia f. domestica*) from Dusseldorf (North-Rhine Westphalia), and in 2016, another Eurasian Blackbird from Giessen (Hesse) tested positive. The molecular results of the wild bird blood samples for each year are highlighted in Table 2. 

The investigated organ samples (brain, liver, spleen or heart as available) from diseased, euthanized or dead-found wild birds, from 2016, from all over Germany, were also analyzed for RNA of WNV and USUV. WNV-specific RNA was not found. In total, together with the results obtained from our cooperation partners (for details see sample collection), we can summarize that for 2016, USUV RNA was detected in organ samples from 73 birds of various species belonging to the orders Passeriformes and Strigiformes (for details see Table 3).

During the period from 2014 to 2016, 58 out of 1825 wild bird sera showed specific neutralizing antibodies against WNV. In 2014, WNV-specific antibodies could be detected in 22 wild birds (Table 4), in 2015, in 20 wild birds (Table 5), and in 2016, in 16 wild birds (Table 6). The titers ranged from 1/10 to 1/240, but were mostly found to be 1/40 or lower. The WNV antibody positive birds were mainly long-distance, partial or short-distance migrants, but also some resident species were affected. The resident and/or partial migrant birds included one Eurasian Magpie (ND_50_ 1/10), three Eurasian Blackbirds (ND_50_ 1/10), two Hooded Crows (ND_50_ 1/10 and 1/20), six Northern Goshawks (ND_50_ 1/10 to 1/15), four City Pigeons (ND_50_ 1/10), one Eurasian Green Woodpecker (ND_50_ 1/20), and one Eurasian Tawny Owl (ND_50_ 1/10). Only low or no USUV-specific antibodies could be detected in these wild birds, thus unspecific cross-reactivity was excluded (for details see Table 4, Table 5 and Table 6).

In the same period, 56 birds with USUV-neutralizing antibodies were detected among the 1825 sera—three of them in 2014, 32 in 2015, and 21 in 2016. The neutralizing titers varied between 1/10 and 1/1920. Most birds belonged to resident species, but also short-distance and particularly, partial migrants were affected. The most frequently affected resident species were Eurasian Blackbirds (11 specimens), but antibodies were also detected in some bird species from the zoological orders, Accipitriformes and Strigiformes (for details see Table 4, Table 5 and Table 6). Furthermore, also two Eurasian Magpies, one House Sparrow, one Great Tit, three Carrion Crows and one Hooded Crow, from the order Passeriformes, which are resident species or partial migrants, were found to have low USUV antibody titers, ranging from 1/10 to 1/15.

In seven wild birds from this study, it was not possible to discriminate between WNV or USUV titers by VNT, because the antibody titers for both viruses were the same or differed only slightly (1–1.5 fold). In detail, these were one Eurasian Blackbird (WNV ND_50_ 1/20; USUV ND_50_ 1/15), one Typical Warbler (WNV ND_50_ 1/10; USUV ND_50_ 1/10), one City Pigeon (WNV ND_50_ 1/15; USUV ND_50_ 1/15) and one White-tailed Eagle (WNV ND_50_ 1/240; USUV ND_50_ 1/120) in 2014, and two Common Buzzards (WNV ND_50_ 1/15; USUV ND_50_ 1/10) and one Eurasian Woodcock (WNV ND_50_ 1/10; USUV ND_50_ 1/10) in 2015. 

The neutralization assay results of all wild bird species between 2014 and 2016 are presented in the Supplemental Table S1.

## 4. Discussion

Wild birds play an important role in virus transmission and spread to new and previously unaffected areas. Every year, migratory birds travel over long distances and successively pass from WNV endemic regions onto new areas [11]. However, WNV viremia in birds usually does not last longer than 6 days, which is too short for long-distance projections, e.g., from the endemic areas in Southern Europe to Northwestern Europe [6,10,40]. Therefore, local bird–mosquito–bird transmission cycles along the routes are required, to allow dispersal. Moreover, other factors (e.g., migratory or climate stress) may prolong viremia in the birds. Finally, persistent subclinical WNV infections lasting over several weeks have also been described for several bird species [9,41]. In the last few years, WNV has been found in horses and birds in different European countries, such as Hungary, Italy and Austria [42,43,44,45]. Due to the geographic proximity, an introduction of WNV to Germany will occur in just a matter of time—the virus is “*ante portas*”.

However, WNV-specific nucleic acids could not be detected in any of the 1902 avian blood samples in the present study. Therefore, there is currently no indication for an autochthonous WNV cycle in resident and migratory birds in Germany. These data are in accordance with earlier WNV studies in birds [19,20,21]. Furthermore, no WNV-specific RNA has been detected in mosquitoes in the different German mosquito surveillance studies to date [46,47].

In contrast, USUV, a closely related flavivirus, was introduced to Europe about twenty years ago, and phylogenetic analyses revealed the occurrence of different USUV lineages: USUV Europe 1–5 and USUV Africa 1–3 [27,33,48]. The virus has been endemic in Germany since 2011/2012, when it caused a massive die-off in Eurasian Blackbirds and Great Grey Owls in the Upper Rhine valley [30]. During the following years (2013–2015), the number of yearly USUV positive cases was low. But in 2016, besides the known USUV epidemic regions in the Upper Rhine valley, a higher occurrence of USUV was found in the Northern parts of North-Rhine Westphalia, up to the border to The Netherlands as well as in the region of Leipzig (Eastern Germany). Phylogenetic analysis of the causative German USUV strains demonstrated the circulation of four lineages, two of which probably have recently spread from Germany to the further affected Western European countries (Belgium, The Netherlands) [32,33]. 

**Figure 2:**
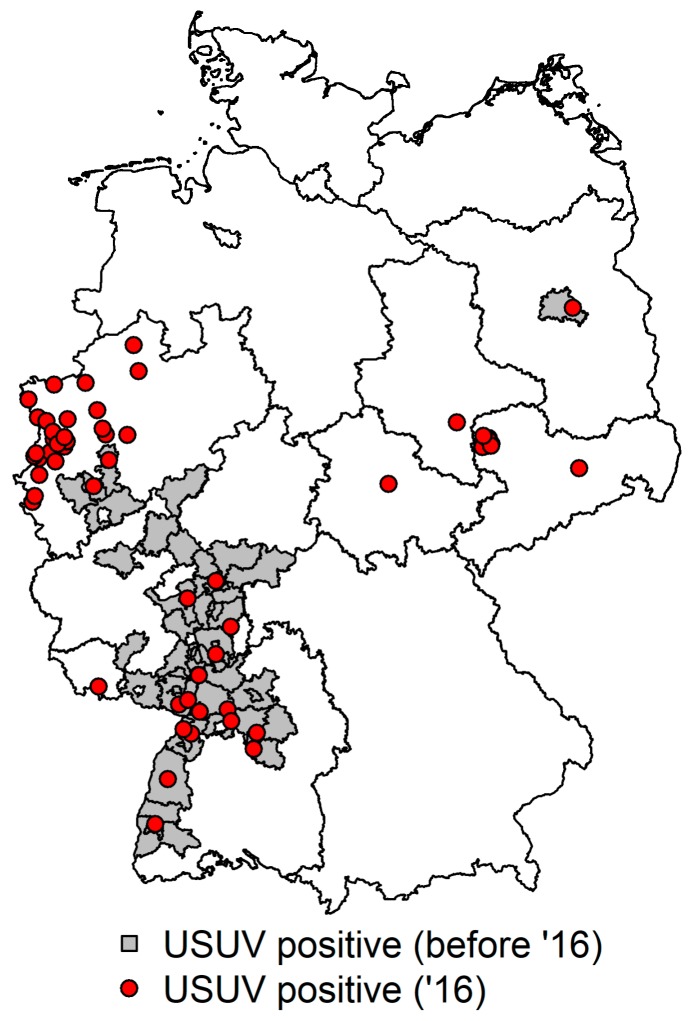
Detection of USUV in dead birds in 2016 (red dot = one USUV positive case/bird in 2016, grey areas = USUV positive areas before 2016).

In the here-presented live bird survey, spanning 2014–2016, only four USUV genome positive animals were detected during blood investigations, which was not surprising as mostly healthy-looking or orphan wild birds, or wild birds found with injuries caused by trauma, were sampled and not primarily birds with neurological symptoms. The positive birds originated from the known USUV epidemic regions (Dusseldorf and Giessen) in Germany. In contrast, the many USUV-diseased or dead-found wild birds identified during dead bird sampling from all over Germany in 2016 illustrate an onward spread and new virus incursions. Several cases in 2016 have been described in publications by Cadar et al. 2017 and Sieg et al. 2017, to date [32,33]. A complete detailed map, showing the origin of all USUV positive dead birds, in 2016, in Germany, is given in Figure 2.

The serological results showed that 58 out of 1825 wild birds, belonging to 10 bird orders, had WNV-neutralizing antibodies, corresponding to 3.18%. Affected birds were mainly long-distance (L), short-distance (S), and partial migrants (P), which probably came in contact with the virus in their overwintering regions in Southern Europe and/or Africa. The number of WNV positive birds as well as the large number of different wild bird species are in line with previous studies [19,20,21]. WNV antibodies were also detected in 18 resident and/or partial migrant bird species (R, P); however, their WNV-neutralizing antibody titers were quite low (ND_50_ 1/10 and 1/20) and the only real resident birds (R) among them were one Eurasian Magpie and one Eurasian Tawny Owl (Table 7). These results are in accordance with previous studies in Germany, but it is still unclear why low WNV-neutralizing antibodies occur in partial migrant and/or resident bird species. An explanation for the WNV seropositivity in raptors, such as the Northern Goshawk, could be that these birds became infected by predating infected migratory birds or scavenging carcasses [8,49]. These wild birds have also been classified as facultative/partial migrants so that the infections may have occurred outside Germany. We were also able to detect very low WNV antibody titers in City Pigeons, which usually also are a resident species, but some specimens also migrate over short distances and may come into contact with infected birds in the neighboring countries. In Greece, Domestic Pigeons have been shown to be suitable sentinels for WNV [50]. Thereby, seroconverted pigeons indicated regions with enzootic virus transmission to warn health authorities at an early stage. Therefore, the number of investigated pigeons should be increased in further studies, to increase sensitivity of the surveillance network. 

WNV seropositive birds were also detected among resident birds in The Netherlands, which according to Lim et al. [49] might indicate that the virus already is circulating in this country. Among the WNV seropositive birds from The Netherlands, there was a large number of Eurasian Coots. Similar findings also were made in different other countries, such as Iran, Spain and the Czech Republic, where a high percentage of the investigated Eurasian Coots demonstrated WNV-neutralizing antibodies [51,52,53]. The species probably plays a special role because they develop a significant antibody response to WNV, thus they may be suitable sentinel animals for WNV. Unfortunately, our sample panel on Eurasian Coots is too small (only four birds in our panel over three years) to draw reliable conclusions. In contrast to Lim et al. [49], who preferentially investigated waterfowls, our focus was on Passerines and birds of prey, which are known to be highly-susceptible to WNV infections; therefore, more than 1000 avian blood samples from these bird groups occurred in our sample panel.

Due to the fact that some partial migrant and resident birds showed neutralizing antibodies against WNV, local circulation of the virus in Germany cannot be excluded. In principle, the potential vector, *Culex pipiens*, is present in Germany, and the susceptibility of indigenous mosquitoes to WNV infection could be demonstrated [54]. Therefore, mosquito sampling in areas where seropositive resident birds have been found seems to be an important surveillance tool to detect local virus circulation. However, despite large scale mosquito screening projects, with over 143 trapping sites, all over Germany, and additional mass-collection of mosquitoes at predisposed places (such as flood areas or big watercourses or rivers), during the main mosquito season, since 2009, WNV-specific RNA has not been detected in mosquitoes so far [47]. The same applies to the detection of WNV-specific RNA in wild birds in this, and in former, studies [20,21]. Therefore, local circulation of the virus so far undetected in mosquito populations, as assumed by Lim et al. 2017 [49] for The Netherlands, is questionable for Germany. In the case of local WNV circulation, an increase of antibody response in resident and/or partial migrant birds should occur over the years, but this was not observed in this study. Instead, we observed a regressive number of birds with specific WNV-neutralizing antibodies from 2014 to 2016, although the number of investigated samples in 2016 was three times as high as in 2014 (see Table 1 and Table 7).

In contrast to the WNV situation, most of the USUV antibody positive birds in our study were resident birds or facultative/partial migrants. The high neutralizing titers varied between 1/60 and 1/1920. The serological investigation showed a relatively small number of USUV antibody positive birds in 2014—neutralizing antibodies could be detected in just three of 248 birds (1.21%). In the following year, 32 of 821 birds (3.9%) and in 2016, 21 of 756 (2.78%) birds, demonstrated neutralizing antibodies against USUV. In 2015 and 2016, we detected significantly more birds with USUV antibodies, with partially high neutralization titers. However, the numbers of USUV antibody positive birds remained low and a comprehensive diffusion of endemic areas was not detectable. In contrast to the situation in Austria, where four to five years after the USUV outbreak, the percentage of seroreactors increased to over 50% and an establishment of herd immunity was seen [55], this has not occurred in Germany yet. Our results show sporadic USUV antibody positive birds in the known USUV epidemic regions. These results are in accordance with the previous study from 2011–2013 by Ziegler et al. 2015 [20]. In other USUV endemic countries, such as Italy, where USUV has been circulating for many years, there is also no evidence for the establishment of herd immunity, as seen in the studies in Austria [56]. Further wild bird investigations will show whether the percentage of USUV positive seroreactors will increase in the future, and if an entry of WNV into the wild bird population takes place in Germany.

## 5. Conclusions

Taken together, the introduction of WNV into Germany along the flyways of migratory birds should be considered a realistic future scenario. A northward spreading tendency of WNV is already apparent, and susceptible vectors and hosts are already present in Germany. Due to the fact that USUV has been endemic in Germany for seven years now, and that WNV and USUV have similar transmission cycles between birds as main amplifying hosts and mosquitoes as vectors, co-circulation as well as overlapping transmission cycles in one area cannot be excluded. Therefore, monitoring activities of the German nationwide wild bird surveillance network for zoonotic arthropod-borne virus infections are essential to reveal a potential future WNV incursion inn enough time, so that suitable public and animal health protection measures can be introduced without delay.

## Figures and Tables

**Table 1:** Total number of bird orders of which blood samples were taken during the monitoring program from 2014 to 2016 in Germany.

Order (-formes)	Year 2014	Year 2015	Year 2016	Total
Passeriformes	80	180	179	439
Accipitriformes/Falconiformes	83	294	265	642
Strigiformes	10	103	96	209
Anseriformes	22	106	39	167
Columbiformes	36	115	117	268
Apodiformes	2	9	12	23
Charadriiformes	2	24	17	43
Ciconiiformes/Pelicaniformes	6	49	22	77
Gruiformes	1	3	2	6
Piciformes	4	28	21	53
Suliformes	2	1	2	5
Cuculiformes	0	2	1	3
Coraciiformes	0	1	0	1
Podicipediformes	0	1	0	1
Psittaciformes	0	1	0	1
Phoenicopteriformes	0	19	0	19
Galliformes	1	2	1	4
Caprimulgiformes	0	0	1	1
**Total**	**249**	**938**	**775**	**1962**

**Table 2:** Results of wild and zoo bird blood samples in quantitative real-time polymerase chain reactions (qRT-PCR) between 2014 and 2016.

Year	West Nile Virus (WNV) qRT-PCR No. Pos./No. Samples Tested	Usutu Virus (USUV) qRT-PCR No. Pos./No. Samples Tested
2014	0/243	1/243
2015	0/892	2/892
2016	0/767	1/767
**Total**	0/1902	**4**/1902

**Table 3:** Dead birds found positive for Usutu virus infection by qRT-PCR in 2016.

Order	Common Name	Scientific Name	Migration Pattern	Housing	No. of USUV RNA Positive Birds
Passeriformes	Eurasian Blackbird	*Turdus merula*	R, P	wild	62
	Common Starling	*Sturnus vulgaris*	R, P, S	wild	1
	Song Trush	*Turdus philomelos*	R, S	wild	1
Strigiformes	Great Grey Owl	*Strix nebulosa*	-	captive	8
	Snowy Owl	*Bubo scandiacus*	-	captive	1
**in Total in 2016**					**73**

R = resident species, P = partial migrant, S = short distance migrant, L = long distance migrant.

**Table 4:** WNV and USUV positive neutralization assay results (positives highlighted in bold) from wild bird serum samples in 2014.

Order	Common Name	Scientific Name	Migration Pattern	No. Samples Tested	WNV Pos. (ND_50_)	USUV Pos. (ND_50_)
Passeriformes	Eurasian Blackbird	*Turdus merula*	R, P	19	1 (20), 1 (40)	**1 (640)**, 1 (15)
	Eurasian Magpie	*Pica pica*	R	2	**1 (10)**	0
	Typical Warbler	*Sylvia* sp.	L	1	1 (10)	1 (10)
	Hooded Crow	*Corvus cornix*	R, P	12	**1 (10)**	0
Accipitriformes	Griffon Vulture	*Gyps fulvus*	zoo bird	2	**1 (30)**	1 (10)
	Northern Goshawk	*Accipiter gentilis*	R, P	10	**4 (10)**	0
	Common Buzzard	*Buteo buteo*	R, P, S	31	**2 (10), 2 (15), 1 (20)**	0
	Western Marsh Harrier	*Circus aeruginosus*	L	3	**1 (240)**	1 (15)
	Red Kite	*Milvus milvus*	(R), S	2	**2 (15)**	0
	White-tailed Eagle	*Haliaeetus albicilla*	R, P	6	1 (240)	1 (120)
	Eurasian Sparrowhawk	*Accipiter nisus*	R, P, S	9	**1 (10)**	0
Falconiformes	European Kestrel	*Falco tinnunculus*	R, P, S	11	**1 (10)**	0
Strigiformes	Eurasian Tawny Owl	*Strix aluco*	R	5	**1 (10)**	0
Columbiformes	Common Wood Pigeon	*Columba palumbus*	R, P, S	13	**1 (15)**	0
	City Pigeon	*Columba livia f. domestica*	R, (P)	21	**1 (10)**, 1 (15)	**1 (10), 1 (20)**, 1 (15)
Anseriformes	Egyptian Goose	*Alopochen aegyptiacus*	zoo bird	2	**1 (10)**	0
Gruiformes	Eurasian Coot	*Fulica atra*	P, S	1	**1 (15)**	0
**Total**				**248**	**22**	**3**

R = resident species, P = partial migrant, S = short distance migrant, L = long distance migrant.

**Table 5:** WNV and USUV positive neutralization assay results (positives highlighted in bold) from wild bird serum samples in 2015.

Order	Common Name	Scientific Name	Migration Pattern	No. Samples Tested	WNV Pos. (ND_50_)	USUV Pos. (ND_50_)
Passeriformes	Eurasian Blackbird	*Turdus merula*	R, P	48	**2 (10)**, 1 (120)	**3 (10), 3 (15), 1 (20), 1 (1920)**
	Eurasian Magpie	*Pica pica*	R	8	0	**1 (10)**
	House Sparrow	*Passer domesticus*	R	13	0	**1 (10)**
	Great Tit	*Parus major*	R, (P)	9	0	**1 (10)**
	Hooded Crow	*Corvus cornix*	R, P	18	0	**1 (15)**
	Carrion Crow	*Corvus corone*	R, P	31	0	**2 (10), 1 (15)**
Accipitriformes	Bearded Vulture	*Gypaetus barbatus*	zoo bird	1	0	**1 (40)**
	Osprey	*Pandion haliaetus*	L	2	**1 (320)**	1 (10)
	Northern Goshawk	*Accipiter gentilis*	R, P	22	**2 (15)**	**1 (40)**
	Common Buzzard	*Buteo buteo*	R, P, S	84	**2 (10), 1 (40)**, 2 (15), 3 *, 1 ^#^	**1 (10)**, 3 (10)
	Eurasian Sparrowhawk	*Accipiter nisus*	R, P, S	12	**1 (15)**	0
	Golden Eagle	*Aquila chrysaetos*	zoo bird	1	0	**1 (10)**
Falconiformes	Red-footed Falcon	*Falco vespertinus*	L	1	**1 (80)**	1 (10)
	European Kestrel	*Falco tinnunculus*	R, P, S	78	**1 (20), 2 (30)**	**1 (10)**, 1 ^#^
Strigiformes	Great Grey Owl	*Strix nebulosa*	zoo bird	4	1 (30)	**1 (480)**
	Eurasian Eagle Owl	*Bubo bubo*	R	14	0	**1 (20)**
	Eurasian Tawny Owl	*Strix aluco*	R	18	1 *	**1 (10), 1 (40)**
	Northern Long-eared Owl	*Asio otus*	R, P, S	19	0, 1 ^#^, 1 *	**1 (10), 1 (20)**
Columbiformes	Common Wood Pigeon	*Columba palumbus*	R, P, S	53	**1 (10)**, 2 *	0
	City Pigeon	*Columba livia f. domestica*	R, (P)	52	**2 (10)**	1 ^#^
Gruiformes	Eurasian Coot	*Fulica atra*	P, S	2	**1 (10)**	0
Apodiformes	Common Swift	*Apus apus*	L	9	**1 (10)**	**1 (10), 1 (60)**
Ciconiiformes/Pelicaniformes	White Stork	*Ciconia ciconia*	L	6	0	**1 (10)**
Charadriiformes	Black-headed Gull	*Larus ridibundus*	R, P, S	1	1 (10)	**1 (30)**
	Gull	*Laridae* sp.	R, P, S, (L)	1	0	**1 (10)**
	Eurasian Woodcock	*Scolopax rusticola*	R, S	16	1 (10)	1 (10)
Piciformes	Great Spotted Woodpecker	*Dendrocopos major*	R, P, (S)	11	**1 (20)**	0
	Eurasian Green Woodpecker	*Picus viridis*	R, (P)	16	**1 (20)**, 1 *	**1 (10)**
**Total**				**821**	**20**	**32**

* Not done because insufficient serum volume for both tests ^#^ Not analyzable because sample is cytotoxic or there were coverings on the cells. R = resident species, P = partial migrant, S = short distance migrant, L = long distance migrant.

**Table 6:** WNV and USUV positive neutralization assay results (positives highlighted in bold) from wild bird serum samples in 2016.

Order	Common Name	Scientific Name	Migration Pattern	No. Samples Tested	WNV Pos. (ND_50_)	USUV Pos. (ND_50_)
Passeriformes	Eurasian Blackbird	*Turdus merula*	R, P	53	**1 (10)**, 1 *, 2 ^#^	**1 (10), 1 (30)**, 1 *
	Eurasian Magpie	*Pica pica*	R	9	0	**1 (10)**
	Hooded Crow	*Corvus cornix*	R, P	10	**1 (20)**	0
	Carrion Crow	*Corvus corone*	R, P	28	**1 (30)**, 1 *	0
Accipitriformes	Long-legged Buzzard	*Buteo rufinus*	zoo bird	2	0	**2 (10)**
	Bearded vulture	*Gypaetus barbatus*	zoo bird	1	1(15)	**1 (30)**
	Common Buzzard	*Buteo buteo*	R, P, S	76	**1 (15)**	**1 (20), 1 (30)**
	Cinereous Vulture	*Aegypius monachus*	zoo bird	1	0	**1 (10)**
	Red Kite	*Milvus milvus*	(R), S	9	**1 (10)**	0
	Rüppell’s Vulture	*Gyps rueppelli*	zoo bird	2	0	**1 (10)**
	European Honey Buzzard	*Pernis apivorus*	L	1	**1 (15)**	0
	White-headed Vulture	*Trigonoceps occipitalis*	zoo bird	2	0	**1 (30)**
Falconiformes	Eurasian Hobby	*Falco subbuteo*	L	3	**1 (80)**	0
	Barbary Falcon	*Falco pelegrinoides*	zoo bird	1	0	**1 (20)**
	European Kestrel	*Falco tinnunculus*	R, P, S	44	**1 (10), 2 (15)**	0
Strigiformes	Short-eared Owl	*Asio flammeus*	L	2	0	**1 (20)**
	Eurasian Tawny Owl	*Strix aluco*	R	14	0	**1 (80)**
	Northern Long-eared Owl	*Asio otus*	R, P, S	19	0	**1 (40)**
Columbiformes	Common Wood Pigeon	*Columba palumbus*	R, P, S	75	**1 (15), 2 (20)**, 1 (15)	**1 (10), 1(30)**
	City Pigeon	*Columba livia f. domestica*	R, (P)	34	**1 (10)**	0
Gruiformes	Eurasian Coot	*Fulica atra*	P, S	1	**1 (30)**	0
Apodiformes	Common Swift	*Apus apus*	L	12	1 (20)	**1 (40)**
Ciconiiformes	Grey Heron	*Ardea cinerea*	R, P, S	12	0	**1 (15)**
	White Stork	*Ciconia ciconia*	L	6	**1 (240)**	1 (20)
Charadriiformes	Black-headed Gull	*Larus ridibundus*	R, P, S	2	0	**1 (10)**
Suliformes	Great Cormorant	*Phalacrocorax carbo*	R, S	2	0	**1 (10)**
**Total**				**756**	**16**	**21**

* Not done because insufficient serum volume for both tests ^#^ Not analyzable because sample is cytotoxic or there were coverings on the cells. R = resident species, P = partial migrant, S = short distance migrant, L = long distance migrant.

**Table 7:** Migration pattern of WNV serologically positive birds (without zoo birds).

Migration Pattern	Year of Samples Collection	Common Name	Scientific Name	WNV Pos. (ND_50_)
L	2014	Western Marsh Harrier	*Circus aeruginosus*	**1 (240)**
L	2015	Osprey	*Pandion haliaetus*	**1 (320)**
L	2015	Common Swift	*Apus apus*	**1 (10)**
L	2015	Red-footed Falcon	*Falco vespertinus*	**1 (80)**
L	2016	European Honey Buzzard	*Pernis apivorus*	**1 (15)**
L	2016	Eurasian Hobby	*Falco subbuteo*	**1 (80)**
L	2016	White Stork	*Ciconia ciconia*	**1 (240)**
P, S	2014	Eurasian Sparrowhawk	*Accipiter nisus*	**1 (10)**
P, S	2015	Eurasian Sparrowhawk	*Accipiter nisus*	**1 (15)**
P, S	2014	European Kestrel	*Falco tinnunculus*	**1 (10)**
P, S	2015	European Kestrel	*Falco tinnunculus*	**1 (20), 2 (30)**
P, S	2016	European Kestrel	*Falco tinnunculus*	**1 (10), 2 (15)**
P, S	2014	Eurasian Coot	*Fulica atra*	**1 (15)**
P, S	2015	Eurasian Coot	*Fulica atra*	**1 (10)**
P, S	2016	Eurasian Coot	*Fulica atra*	**1 (30)**
P, S	2016	Carrion Crow	*Corvus corone*	**1 (30)**
(R), S	2014	Red Kite	*Milvus milvus*	**2 (15)**
(R), S	2016	Red Kite	*Milvus milvus*	**1 (10)**
R, P, S	2014	Common Buzzard	*Buteo buteo*	**2 (10), 2 (15), 1 (20)**
R, P, S	2015	Common Buzzard	*Buteo buteo*	**2 (10), 1 (40)**
R, P, S	2016	Common Buzzard	*Buteo buteo*	**1 (15)**
R, P, S	2014	Common Wood Pigeon	*Columba palumbus*	**1 (15)**
R, P, S	2015	Common Wood Pigeon	*Columba palumbus*	**1 (10)**
R, P, S	2016	Common Wood Pigeon	*Columba palumbus*	**1 (15), 2 (20)**
R, P, S	2015	Great Spotted Woodpecker	*Dendrocopos major*	**1 (20)**
R, P	2014	Hooded Crow	*Corvus cornix*	**1 (10)**
R, P	2016	Hooded Crow	*Corvus cornix*	**1 (20)**
R, P	2015	Eurasian Blackbird	*Turdus merula*	**2 (10)**
R, P	2016	Eurasian Blackbird	*Turdus merula*	**1 (10)**
R, P	2014	Northern Goshawk	*Accipiter gentilis*	**4 (10)**
R, P	2015	Northern Goshawk	*Accipiter gentilis*	**2 (15)**
R, (P)	2014	City Pigeon	*Columba livia f. domestica*	**1 (10)**
R, (P)	2015	City Pigeon	*Columba livia f. domestica*	**2 (10)**
R, (P)	2016	City Pigeon	*Columba livia f. domestica*	**1 (10)**
R, (P)	2015	Eurasian Green Woodpecker	*Picus viridis*	**1 (20)**
R	2014	Eurasian Magpie	*Pica pica*	**1 (10)**
R	2014	Eurasian Tawny Owl	*Strix aluco*	**1 (10)**
**Total**				**56**

R = resident species, P = partial migrant, S = short distance migrant, L = long distance migrant.

## Supplementary Materials

The following are available online at http://www.mdpi.com/1660-4601/15/1/171/s1. Table S1: WNV and USUV neutralization assay results from all wild bird blood samples between 2014 and 2016.
