# Supplementary material for: West Nile Virus and Usutu Virus Monitoring of Wild Birds in Germany
Source: Int J Environ Res Public Health. 2018 Jan 22;15(1):171. doi: 10.3390/ijerph15010171 (PMC5800270; doi:10.3390/ijerph15010171)
Supplement: Supplementary file 1 [file ijerph-15-00171-s001.pdf]

Table S1. WNV and USUV neutralization assay results from all wild bird blood samples between 2014 and 2016. Positive samples are highlighted in bold, neutralization titres in brackets.

Order	Common Name	Scientific Name	Migration Pattern	No. Samples Tested	WNV pos. (ND_{50})	USUV pos. (ND_{50})
Passeriformes	Carrion Crow	*Corvus corone*	R, P	1	0	0
	Eurasian Blackbird	*Turdus merula*	R, P	120	**3 (10)**, 1 (20), 1 (40) 1 (120), 1 *, 2 [#]	**4 (10), 3 (15), 1 (20), 1 (30), 1 (640), 1 (1920)**, 1 (15), 1 *
	Tomtit	*Cyanistes caeruleus*	R	4	0, 1*	0
	Common Chaffinch	*Fringilla coelebs*	R, P	3	0	0
	European Goldfinch	*Carduelis carduelis*	R, P	1	0	0
	Western Jackdaw	*Coloeus monedula*	S	7	0	0
	Eurasian Bullfinch	*Pyrrhula pyrrhula*	R, P	2	0	0
	Thrush	*Turdus sp.*	S, L	17	0	0
	Eurasian Jay	*Garrulus glandarius*	R, P	12	0	0
	Eurasian Magpie	*Pica pica*	R	19	**1 (10)**	**2 (10)**
	Eurasian Siskin	*Carduelis spinus*	S	1	0	0
	Willow Warbler	*Phylloscopus trochilus*	L	1	0	0
	Finch	*Fringilla sp.*	R, P, S	1	0	0
	Garden Warbler	*Sylvia borin*	L	1	0	0
	Common Redstart	*Phoenicurus phoenicurus*	L	1	0	0
	Yellowhammer	*Emberiza citrinella*	R, P, S	5	0	0
	Spotted Flycatcher	*Muscicapa striata*	L	1	0	0
	Typical Warbler	*Sylvia sp.*	L	1	1 (10)	1 (10)
	Black Redstart	*Phoenicurus ochruros*	P, S	3	0	0
	House Sparrow	*Passer domesticus*	R	24	0, 2 *	**1 (10)**
	Grosbeak	*Coccothraustes coccothraustes*	S	8	0	0
	Eurasian Nuthatch	*Sitta europaea*	R	2	0	0
	Great Tit	*Parus major*	R, (P)	11	0	**1 (10)**
	Common Raven	*Corvus corax*	R, P	6	0	0
	Crow	*Corvus sp.*	R, P	9	0	0
	Common House Martin	*Delichon urbicum*	L	11	0	0
	Eurasian Blackcap	*Sylvia atricapilla*	S, L	2	0	0
	Hooded Crow	*Corvus cornix*	R, P	40	**1 (10), 1 (20)**	**1 (15)**
	Carrion Crow	*Corvus corone*	P, S	72	**1 (30)**, 1 *	**2 (10), 1 (15)**
	Barn Swallow	*Hirundo rustica*	L	5	0	0

	European Robin	*Erithacus rubecula*	P	6	1 *	0
	Rook	*Corvus frugilegus*	R, P, S	8	1 [#], 2 *	1 [#]
	Song Thrush	*Turdus philomelos*	R, S	9	1 *	0
	Eurasian Tree Sparrow	*Passer montanus*	R	2	0	0
	Common Starling	*Sturnus vulgaris*	R, P, S	8	0	0
	Coal Tit	*Periparus ater*	R, P	1	0	0
	Fieldfare	*Turdus pilaris*	S	7	0	0
	Common Chiffchaff	*Hylloscopus collybita*	S, L	1	0	0
Accipitriformes	Long-legged Buzzard	*Buteo rufinus*	zoo bird	4	0	**2 (10)**
	Black-chested Buzzard-eagle	*Geranoaetus melanoleucus*	zoo bird	2	0	0
	Bearded Vulture	*Gypaetus barbatus*	zoo bird	2	1 (15)	**1 (30), 1 (40)**
	Osprey	*Pandion haliaetus*	L	4	**1 (320)**	1 (10)
	Griffon Vulture	*Gyps fulvus*	zoo bird	11	**1 (30)**	1(10)
	Northern Goshawk	*Accipiter gentilis*	R, P	47	**4 (10), 2 (15)**	**1 (40)**
	Harpy Eagle	*Harpia harpyja*	zoo bird	1	0	0
	Harris hawk X Red-tailed Hawk Hybrid		zoo bird	1	0	0
	Imperial Eagle	*Aquila heliaca*	zoo bird	4	0	0
	Hen Harrier	*Circus cyaneus*	R, S	1	0	0
	Ferruginous hawk	*Buteo regalis*	zoo bird	4	0	0
	King Vulture	*Sarcoramphus papa*	zoo bird	2	0	0
	Common Buzzard	*Buteo buteo*	R, P, S	191	**4 (10), 3 (15), 1 (20), 1 (40),** 2 (15), 3 *, 1 [#]	**1 (10), 1 (20), 1 (30)**, 3 (10)
	Cinereous Vulture	*Aegypius monachus*	zoo bird	2	0	**1 (10)**
	Steller's sea Eagle	*Haliaeetus pelagicus*	zoo bird	1	0	0
	Western Marsh Harrier	*Circus aeruginosus*	L	6	**1 (240)**	1 (15)
	Red Kite	*Milvus milvus*	(R), S	22	**1 (10), 2 (15)**, 1 [#]	0
	Red-tailed Hawk	*Buteo jamaicensis*	zoo bird	2	0	0
	African Fish Eagle	*Haliaeetus vocifer*	zoo bird	4	0	0
	Black Kite	*Milvus migrans*	L	7	0	0
	White-tailed Eagle	*Haliaeetus albicilla*	R, P	41	1 (240)	1 (120)
	Eurasian Sparrowhawk	*Accipiter nisus*	R, P, S	31	**1 (10), 1 (15)**	0
	Rüppell's Vulture	*Gyps rueppelli*	zoo bird	3	0	**1 (10)**
	Golden Eagle	*Aquila chrysaetos*	zoo bird	7	0	**1 (10)**
	Steppe Eagle	*Aquila nipalensis*	zoo bird	4	0	0
	Bald Eagle	*Haliaeetus leucocephalus*	zoo bird	12	0	0

	Europ. Honey Buzzard	*Pernis apivorus*	L	7	**1 (15)**	0
	White -headed Vulture	*Trigonoceps occipitalis*	zoo bird	3	0	**1 (30)**
	Harris -Hawk	*Parabuteo unicinctus*	zoo bird	13	0	0
Falconiformes	Eurasian Hobby	*Falco subbuteo*	L	7	**1 (80)**	0
	Striated Caracara	*Phalcoboenus australis*	zoo bird	2	0	0
	Gyrfalcon	*Falco rusticolus*	zoo bird	5	0	0
	Gyrfalcon X Saker Falcon Hybrid		zoo bird	1	0	0
	Gyrfalcon X Peregrine Falcon Hybrid		zoo bird	1	0	0
	Lanner Falcon	*Falco biarmicus*	zoo bird	3	0	0
	Merlin	*Falco columbarius*	zoo bird	1	0	0
	Red-footed Falcon	*Falco vespertinus*	L	1	**1 (80)**	1 (10)
	Barbary Falcon	*Falco pelegrinoides*	zoo bird	1	0	**1 (20)**
	Saker Falcon	*Falco cherrug*	zoo bird	7	0	0
	Southern Crested Caracara	*Caracara plancus*	zoo bird	4	0	0
	European Kestrel	*Falco tinnunculus*	R, P, S	133	**2 (10), 2 (15), 1 (20), 2 (30)**	**1 (10)**, 1 #
	Peregrine Falcon	*Falco peregrinus*	R, P	24	0	0
Strigiformes	Great Horned Owl	*Bubo virginianus*	zoo bird	2	0	0
	Great Grey Owl	*Strix nebulosa*	zoo bird	12	1 (30)	**1 (480)**
	Owl	*Strigidae (Fam.)*	R, P, S, L	1	0	0
	Spotted Eagle-owl	*Bubo africanus*	zoo bird	9	0	0
	Ural Owl	*Strix uralensis*	zoo bird	7	0	0
	Boreal Owl	*Aegolius funereus*	R, P	1	1 *	0
	Common Barn Owl	*Tyto alba*	R, P	40	0, 1 *	0
	Snowy Owl	*Bubo scandiacus*	zoo bird	15	0	0
	Northern Hawk Owl	*Surnia ulula*	zoo bird	2	0	0
	Eurasian Pygmy Owl	*Glaucidium passerinum*	R	1	0	0
	Little Owl	*Athene noctua*	R	15	0	0
	Short-eared Owl	*Asio flammeus*	L	2	0	**1 (20)**
	Eurasian Eagle Owl	*Bubo bubo*	R	24	0	**1 (20)**
	Eurasian Tawny Owl	*Strix aluco*	R	37	**1 (10)**, 1 *	**1 (10), 1 (40), 1 (80)**
	Northern Long-eared Owl	*Asio otus*	R, P, S	39	1 #, 1 *	**1 (10), 1 (20), 1 (40)**
Columbiformes	Common Wood Pigeon	*Columba palumbus*	R, P, S	141	**1 (10), 2 (15), 2 (20)**, 1 (15), 2 *	**1 (10), 1 (30)**
	City Pigeon	*Columba livia f. domestica*	R, (P)	119	**4 (10)**, 1 (15), 1 *	**1 (10), 1 (20)**, 1 (15), 1 #
	Eurasian Collared Dove	*Streptopelia decaocto*	R, (P)	2	0	0
Anseriformes	Common Shelduck	*Tadorna tadorna*	S	1	0	0

	Greater white-fronted Goose	*Anser albifrons*	S	1	0	0
	Duck	*Anas platyrhynchos*	R, P, S	27	0	0
	Graylag Goose	*Anser anser*	R, P, S	4	0	0
	Northern Mallard Duck	*Anas platyrhynchos*	R, P, S	1	0	0
	Mute Swan	*Cygnus olor*	R, P, S	58	0	0
	Canada Goose	*Branta canadensis*	zoo bird	1	0	0
	Pink-footed Goose	*Anser brachyrhynchus*	zoo bird	1	0	0
	Egyptian Goose	*Alopochen aegyptiacus*	zoo bird	6	**1 (10)**	0
	Swan	*Cygnus sp.*	R, P, S	4	0	0
	Swan Goose	*Anser cygnoides*	zoo bird	1	0	0
	Whooper Swan	*Cygnus cygnus*	S	2	0	0
	Common Scoter	*Melanitta nigra*	S	2	0	0
Gruiformes	Eurasian Coot	*Fulica atra*	P, S	4	**1 (10), 1 (15), 1 (30)**	0
	Common Moorhen	*Gallinula chloropus*	R, P, S	2	0	0
Apodiformes	Common Swift	*Apus apus*	L	23	**1 (10)**, 1 (20)	**1 (10), 1 (40), 1 (60)**
Caprimulgiformes	European Nightjar	*Caprimulgus europaeus*	L	1	0	0
Ciconiformes	Northern Gannet	*Morus bassanus*	zoo bird	1	0	0
Pelicaniformes	Grey Heron	*Ardea cinerea*	R, P, S	27	1 *	**1 (15)**
	Heron	*Ardeidae (Fam.)*	R, P, S, L	2	0	0
	Black Stork	*Ciconia nigra*	L	2	0	0
	Great Egret	*Ardea alba*	zoo bird	1	0	0
	White Stork	*Ciconia ciconia*	L	13	**1 (240)**	**1 (10)**, 1 (20)
Charadriiformes	Sandwich Tern	*Thalasseus sandvicensis*	S, L	1	0	0
	Lesser Black-backed Gull	*Larus fuscus*	P, S, L	1	0	0
	Black-headed Gull	*Larus ridibundus*	R, P, S	3	1 (10)	**1 (10), 1 (30)**
	Gull	*Laridae (Fam.)*	R, S, L	1	0	**1 (10)**
	European Herring Gull	*Larus argentatus*	R, S	8	0, 2 *	0
	Common Gull	*Larus canus*	R, S	4	0	0
	Eurasian Woodcock	*Scolopax rusticola*	R, S	25	1 (10), 1 *	1 (10)
Coraciiformes	Common Kingfisher	*Alcedo atthis*	R, S	1	0	0
Cuculiformes	Common Cuckoo	*Cuculus canorus*	L	3	1 *	0
Galliformes	Common Pheasant	*Phasianus colchicus*	R	1	0	0
	Rock Partridge	*Alectoris graeca*	R	1	0	0
	Common Quail	*Coturnix coturnix*	S, L	1	0	0
Piciformes	Great Spottet Woodpecker	*Dendrocopos major*	R, P, (S)	25	**1 (20)**	0

	Eurasian Green Woodpecker	*Picus viridis*	R, (P)	25	**1 (20)**, 1 *	**1 (10)**
	Black Woodpecker	*Dryocopus martius*	R, P	2	0	0
Podicipediformes	Great Crested Grebe	*Podiceps cristatus*	R	1	0	0
Psittaciformes	Rose-ringed Parakeet	*Psittacula krameri*	zoo bird	1	0	0
Suliformes	Great Cormorant	*Phalacrocorax carbo*	R, S	5	1 *	**1 (10)**
**Total**				**1825**	**58**	**56**

* Not done because insufficient serum volume for both tests # Not analyzable because sample is cytotoxic or coverings on the cells. R = resident species, P = partial migrant, S = short distance migrant, L = long distance migrant.
